# Granulocyte-colony stimulating factor-producing malignant phyllodes tumor of the breast: a rare case

**DOI:** 10.1186/s40792-021-01113-x

**Published:** 2021-01-15

**Authors:** Kimihisa Mizoguchi, Kazuhisa Kaneshiro, Makoto Kubo, Yoshihiko Sadakari, Yoshizo Kimura, Koichi Higaki, Toshiro Ogata, Masahiko Taniguchi

**Affiliations:** 1 Department of Surgery, St. Mary’s Hospital, 422, Tsubukuhonmachi, Kurume, Fukuoka 830-8543 Japan; 2 Department of First Surgery, Kyusyu University Hospital, 3-1-1, Maidashi, Fukuoka, Fukuoka 812-8582 Japan; 3 Department of Pathology, St. Mary’s Hospital, 422, Tsubukuhonmachi, Kurume, Fukuoka 830-8543 Japan

**Keywords:** Granulocyte-colony stimulating factor, G-CSF receptor, Breast cancer, Malignant phyllodes tumor

## Abstract

**Background:**

Granulocyte-colony stimulating factor (G-CSF)-producing tumors can cause leukocytosis despite an absence of infection. G-CSF-producing tumors have been reported in various organs such as the lung, esophagus, and stomach but rarely in the breast. We report a case of G-CSF-producing malignant phyllodes tumor of the breast.

**Case presentation:**

An 84-year-old woman visited our hospital complaining of a lump in her left breast without fever and pain. Laboratory tests revealed elevated white blood cell (WBC) count and G-CSF levels. A malignant tumor of the breast was diagnosed by core needle biopsy. We performed a total mastectomy and sentinel lymph node biopsy. The tumor was identified as a G-CSF-producing malignant phyllodes tumor. Within 7 days after surgery, the patient’s WBC count and G-CSF level had decreased to normal levels. She is alive without recurrence 13 months after surgery.

**Conclusions:**

We encountered a rare case of G-CSF-producing malignant phyllodes tumor of the breast. PET–CT revealed diffuse accumulation of FDG in the bone. Phyllodes tumors need to be differentiated from bone metastasis, lymphoma, and leukemia. We must be careful to not mistake this type of tumor for bone marrow metastasis.

## Background

Granulocyte-colony stimulating factor (G-CSF)-producing tumors have been shown to cause leukocytosis despite the absence of infection. [1] G-CSF-producing tumors have been reported in various organs such as the lung, bladder, and stomach but rarely in the breast [2–4]. G-CSF-producing carcinomas progress rapidly and have poor prognosis. [5] We report a case of G-CSF-producing malignant phyllodes tumor of the breast.

## Case presentation

**Fig. 1:**
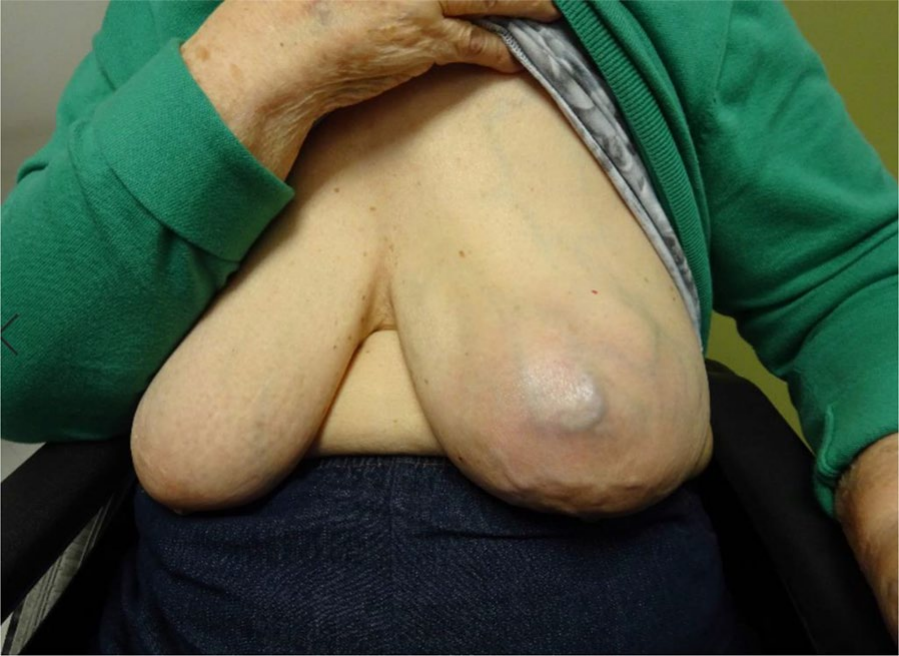
Photograph of the left breast showing a large tumor. The tumor measures approximately 8 cm in size and occupies the entire left breast

**Fig. 2:**
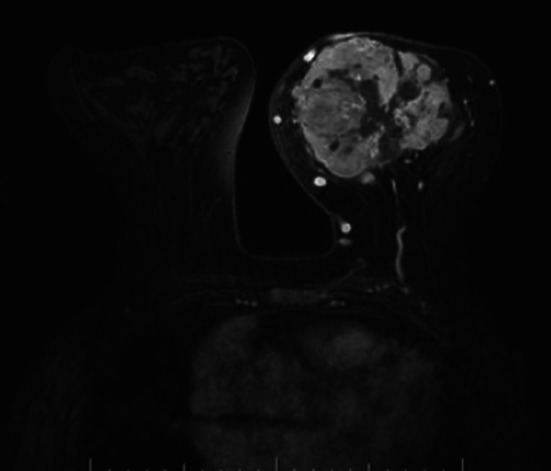
Contrast-enhanced magnetic resonance imaging revealing a large lobulated mass. No apparent infiltration to the pectoral major muscle can be observed

**Fig. 3:**
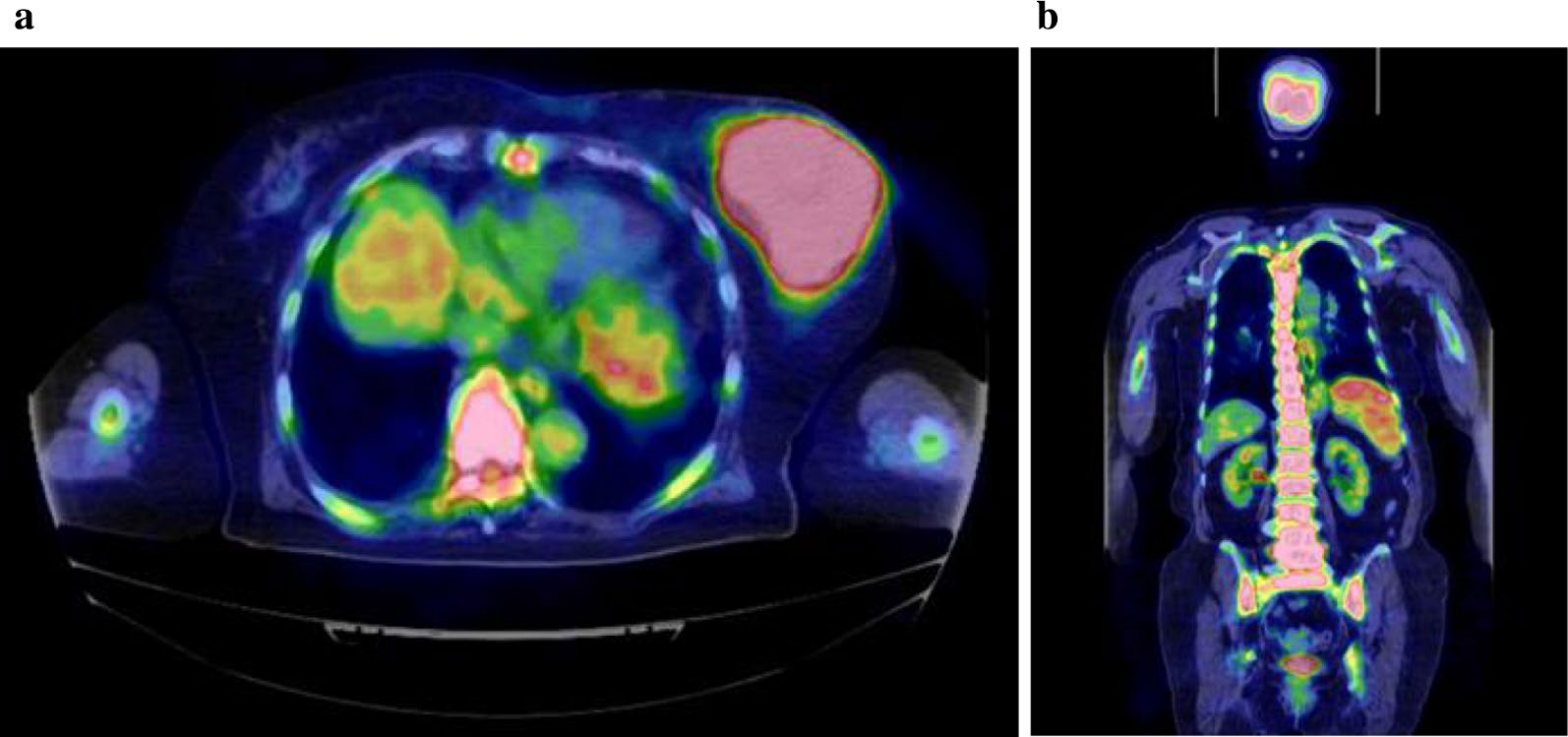
Fluorodeoxyglucose (FDG) positron emission tomography–computed tomography (PET–CT) of the left breast. **a** PET–CT reveals no distant metastasis, although mild-to-moderate homogeneity is seen. **b** FDG uptake is detected in the spine, pelvic bone, and long bones, suggesting bone marrow hyperactivity

**Fig. 4:**
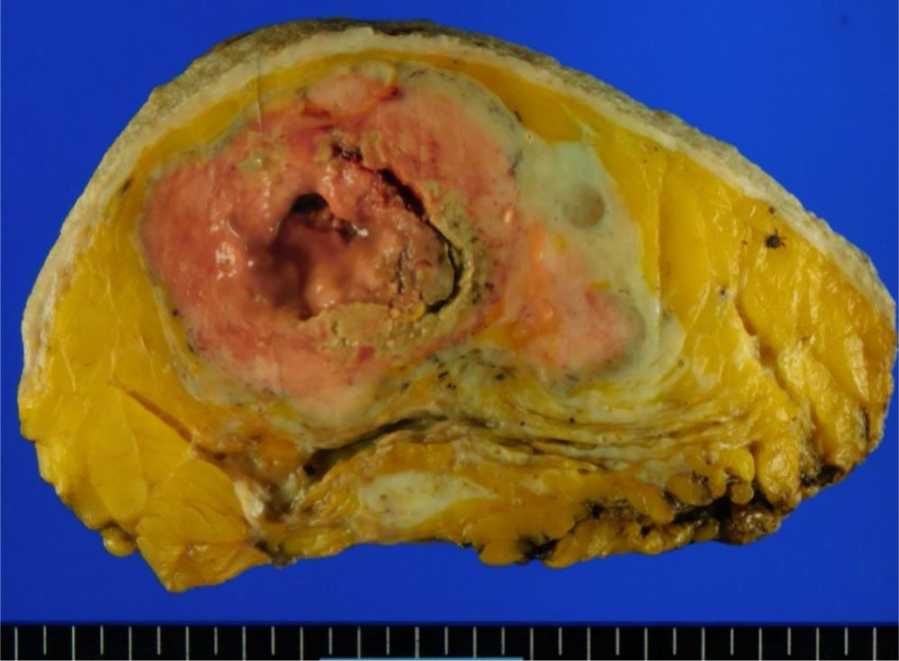
Macroscopic findings showing a large and lobulated tumor

**Fig. 5:**
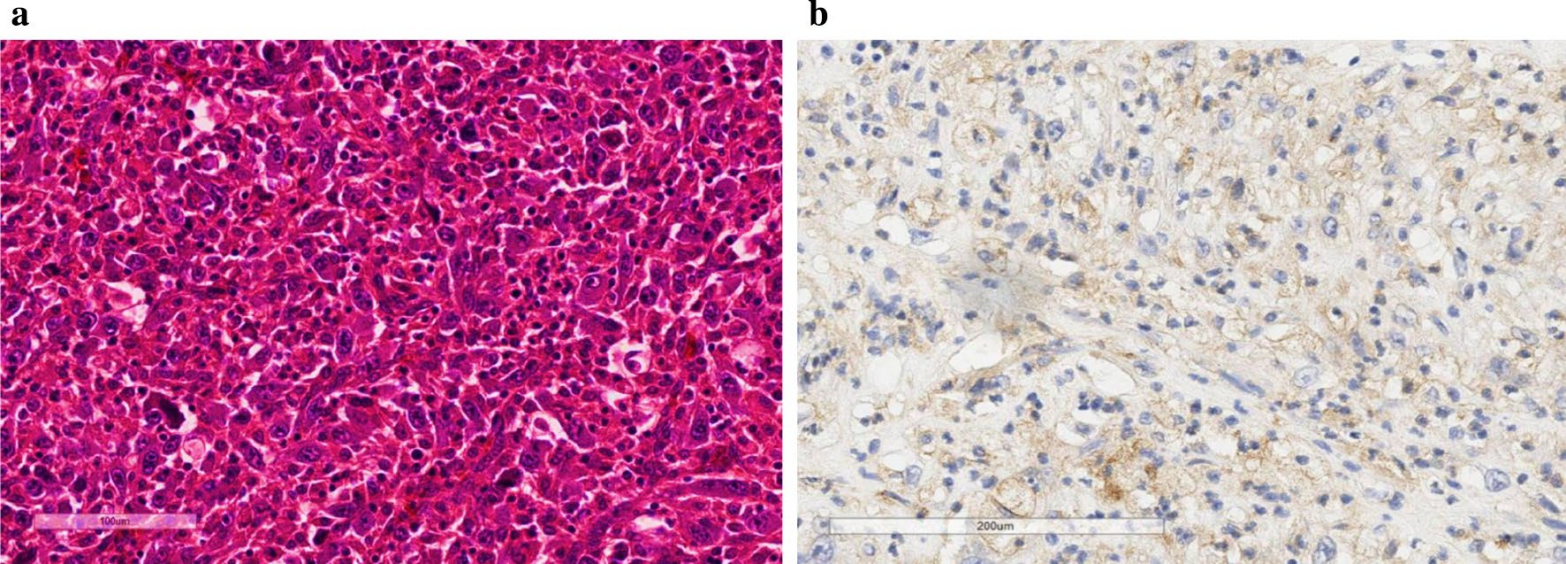
Histologic examination of the excised tumor. **a** H&E stain reveals many poorly differentiated and highly atypical cells. Tumors mainly consist of spindle-shaped to polygonal atypical high-grade stromal cells that grow into sheets while forming multinucleated giant cells. **b** Immunostaining of the tumor cells is positive for G-CSF

An 84-year-old woman suddenly noticed a lump in her left breast, so she visited our hospital. Physical examination revealed a large tumor with redness approximately 8 cm in the maximal dimension, which was occupying the entire left breast. Laboratory tests showed an elevated white blood cell (WBC) count (49,760 cells/μL with 87% neutrophils) without fever and a high G-CSF level (498 pg/mL). Tumor markers were normal (CEA 1.0 ng/mL, CA15-3 13.8 U/mL). Mammography revealed a large, high-density mass in her left breast (Fig. 1). Ultrasound examination revealed a lobulated, inhomogeneous, blood-rich mass. Contrast-enhanced magnetic resonance imaging revealed a large lobulated mass without apparent infiltration to the pectoral major muscle (Fig. 2). Computed tomography showed no enlarged axillary lymph nodes. Fluorodeoxyglucose (FDG) positron emission tomography–computed tomography (PET–CT) revealed no distant metastasis, although mild-to-moderate and homogeneous FDG uptake were detected in the spine, pelvic bone, and long bones, suggesting bone marrow hyperactivity (Fig. 3). She did not present splenomegaly. Histological diagnosis was difficult to confirm using core needle biopsy. These results suggested that this tumor was malignant, such as carcinoma, carcinosarcoma, or sarcoma of the breast. Bone marrow biopsy showed no evidence of bone metastasis. According to the standard treatment of breast cancer, we performed total mastectomy and sentinel lymph node biopsy. There was no lymph node metastasis. Macroscopic findings of excised specimens showed a huge and lobulated tumor, which was 8 cm in size (Fig. 4). The tumor cells showed no expression of the human epithelial growth factor receptor 2 (HER2). Histologic examination with hematoxylin and eosin (H&E) stain revealed many poorly differentiated and highly atypical cells (Fig. 5a). Immunostaining of the tumor cells was negative for AE1/AE3, EMA, and desmin. In addition, it was positive for G-CSF, indicating that the tumor was a G-CSF-producing malignant phyllodes tumor of the breast (Fig. 5a). After surgery, her WBC count had decreased to a normal level within 7 days. She was discharged 10 days after surgery and treated with no adjuvant therapy. She is alive without recurrence 13 months after surgery.

## Discussion

In 1951, Fahey presented the possibility that tumors themselves can produce substances capable of stimulating the bone marrow [6]. Asano et al. first reported the production of G-CSF by a carcinoma in 1977 [7]. Since then, G-CSF-producing tumors have been reported mainly in lung, bladder, and gastric cancers [2–4]. We searched PubMed and Google Scholar using the keywords “G-CSF production” and “breast cancer”. As a result, there were only two well-reported cases of G-CSF-producing breast cancer [5, 8]. According to Anano’s report, the diagnostic criteria for a G-CSF-producing tumor are extreme leukocytosis, elevated G-CSF activity, a decrease in the WBC count after tumor resection, and proof of G-CSF production in the tumor [7]. In our patient, laboratory tests showed an elevated WBC count. However, there was no redness and swelling in her left breast, and she was afebrile. Serum G-CSF level was high (498 pg/mL). Her WBC count and G-CSF had declined to a normal level within 7 days. Thus, our patient met the diagnostic criteria.

Histologically, G-CSF-producing tumors are often poorly differentiated or undifferentiated [8]. Currently, there are no clear guidelines for the therapy of G-CSF-producing tumors [9] and the therapeutic strategy is usually selected based on the primary organ that is affected [10]. However, the prognosis of G-CSF-producing tumors is poor, regardless of the primary organ affected, and average survival time is only a few months [8].

PET–CT showed multiple uptakes in the primary tumor, brain, urinary system, and bones in the patient. Unlike bone metastasis, uptakes in the bones were diffusely distributed within the spine, pelvic, and long bones. This is a characteristic finding of G-CSF-producing tumors. In 1998, Sugawara reported FDG uptake in the bone marrow when G-CSF preparations were administered to patients undergoing chemotherapy [12]. The accumulation of FDG in PET–CT reflects the glucose metabolism of cells. It has been suggested that G-CSF increases the hematopoietic activity of the granulocyte system of the bone marrow and enhances glucose metabolism [13]. In our patient, we performed a bone marrow biopsy to rule out metastasis and leukemia, which was confirmed.

Breast tumors mainly consist of spindle-shaped to polygonal atypical high-grade stromal cells that grow into sheets while forming multinucleated giant cells. The sarcoma component of the tumor was negative for AE1/AE3 and EMA; therefore, a diagnosis of metaplastic carcinoma was negative. The tumor had atypical ductile hyperplasia showing hyperplasia at the same time as mesenchymal tumor cells. We, therefore, determined it to be a phyllodes tumor. In addition, the nucleoli were clearly visible in the tumor cells. The patient was diagnosed with a malignant phyllodes tumor. Neutrophil infiltration was observed between the tumor cells, which was consistent with the diagnosis of a G-CFS-producing tumor.

Phyllodes tumors account for 0.3–1.0% of breast tumors [14]. They are classified into benign, borderline, and malignant according to histopathologic features [12]. It is often difficult to distinguish benign from malignant phyllodes tumors from other benign tumors such as fibroadenomas before surgery [15]. Approximately 10–15% of phyllodes tumors are malignant [10]. Malignant phyllodes tumors are characterized by a typical rapid growth and reported to cause local recurrence at a rate of 20–65% [15, 16]. Rapid surgery with proper margin to determine an accurate diagnosis and careful follow-up after surgery are important [17].

## Conclusions

We treated a patient with G-CSF-producing malignant phyllodes tumor of the breast. It was a triple-negative type malignant tumor that was mainly composed of atypical stromal cells as in the past two reported cases [5, 11]. Including this case, a G-CSF-producing tumor has presented as a malignant tumor mainly composed of stromal cells. In addition, PET–CT revealed diffuse accumulation of FDG in the bone. Phyllodes tumors need to be differentiated from bone metastasis, lymphoma, and leukemia. We must be careful to not mistake this type of tumor for bone marrow metastasis.

## Data Availability

The data supporting the conclusions of this article are included within the article.

## Abbreviations

FDG: Fluorodeoxyglucose
G-CSF: Granulocyte-colony stimulating factor
H&E: Hematoxylin and eosin
HER2: Human epithelial growth factor receptor 2
PET–CT: Positron emission tomography–computed tomography
WBC: White blood cell
